# Family resources and school performance: an analysis of associated factors in middle school adolescents

**DOI:** 10.1590/2317-1782/20212021058

**Published:** 2022-01-05

**Authors:** Rebeca Mota Cabral e Silva, Graziela Nunes Alfenas Fernandes, Andrezza Gonzalez Escarce, Stela Maris Aguiar Lemos

**Affiliations:** 1 Departamento de Fonoaudiologia, Faculdade de Medicina, Universidade Federal de Minas Gerais – UFMG - Belo Horizonte (MG), Brasil.

**Keywords:** Adolescent, Academic Performance, Family, Social Class, Speech, Language and Hearing Sciences

## Abstract

**Purpose:**

To verify the association between family resources, sociodemographic aspects, and school performance in middle school students.

**Methods:**

Observational, analytical, cross-sectional study with a probabilistic sample stratified by sex, age, and grade in school. A total of 124 adolescents of both sexes, aged 11 to 14 years, enrolled in a private middle school participated in the study. We sent to their parents/guardians an online form with the Home Environment Resources Scale (HER), Brazilian Economic Classification Criteria (CCEB), and sociodemographic questions. The school performance was furnished by the institution. We used frequency distribution analysis of categorical variables and measures of central tendency and dispersion of the continuous ones. We used the Mann-Whitney, Kruskal-Wallis, and Spearman correlation coefficient tests and set the significance level at p ≤ 0.05.

**Results:**

Most of the sample was 11 years old, females, sixth graders, with very good school performance, belonging to social class A, and whose householder had a bachelor’s degree. Age and grade in school were associated with interaction with the parents, “family-school connection”, and total HER score. The “proximal processes” had a moderate positive correlation with outings and toys. There was a moderate positive correlation between total HER score and “predictable activities that indicate stability”. The “proximal processes” had a strong positive correlation with “family-school connection”. There was a weak positive correlation between books available at home and school performance.

**Conclusion:**

The family resources were related to the adolescents’ school performance.

## INTRODUCTION

School performance is built upon a series of elements. Considering that poor school performance does not result only from what an adolescent does at school, we can approach academic success as the outcome of multiple variables. Factors related to the student and the various contexts to which they belong – family, social, cultural, institutional, political, and economic – have been pointed out as agents that influence the results at school^(1)^. The environment can either negatively or positively influence a person’s development, and the family is seen as an essential context in children’s and adolescents’ learning processes^(2,3)^. Studies indicate that the parents’ involvement with their children’s school activities is one of the elements that increase the students’ interest in such activities, besides revealing how important the parents consider education^(4-6)^.

The interaction between family and school draws researchers’ attention^(4,7)^ because the parents’ involvement with education has been seen as an essential element to good academic performance^(7)^. The phenomena related to education and to the family-school relationship represent an expressive portion of what teachers, administrators, educators, and education specialists produce in their concern with such a relationship. This interest arises from the need for answering important issues regarding the extent of the effects of this interaction, the unfolding of its possible benefits or harms, and the role played by the family-school connection in the student’s comprehensive education^(7)^. A systematic review aimed to find the state of the art on this topic – family-school relationship in Brazil – and selected studies demonstrating that a good school-parents relationship may even prevent dysfunctional behaviors^(8)^.

School experiences can interfere with the person’s future and path in life. Accordingly, school failure helps increase their vulnerability and lower their self-esteem^(9)^. Understanding factors that influence poor performance in different contexts helps develop strategies to address poor school performance.

The objective of this study was to verify the association between home resources – based on data from the Home Environment Resources Scale (HER) – sociodemographic aspects, and school performance in middle school students.

## METHODS

This is an observational, analytical, cross-sectional study with a probabilistic sample stratified by sex, age, and grade in school. Adolescents of both sexes, aged 11 to 14 years, participated in the study. They were enrolled in a private middle school in Belo Horizonte, Minas Gerais, Brazil. The parents/guardians and adolescents who agreed to participate respectively signed the informed consent form and the informed assent form.

This study complied with the human research guidelines and norms established in Resolution 466, from December 12, 2012, and was approved by the institution’s Research Ethics Committee under evaluation report number 2.422.795.

The inclusion criteria were students 11 to 14 years old enrolled in the middle school where this research took place. Adolescents with cognitive, neurological, or psychiatric impairments that hindered them from participating in the research (based on observing their behavior when the questionnaires were administered) were excluded from the study.

We followed these procedures to achieve the research objective:

- Home Environment Resources Scale (HER)^(10)^: this questionnaire assesses aspects from the home environment that might interfere with school performance. It has 10 multiple-choice questions categorized into three broad domains: “resources that promote proximal processes”, “predictable activities that indicate some degree of stability of family life”, and “parental practices that promote family-school connection”. The “resources that promote proximal processes” encompass areas and activities such as the parents’ participation in stimulating experiences, opportunities to interact with the parents, and access to scheduled learning activities. The “predictable activities that indicate some degree of stability of family life” include routine activities, family time regularly spent together, and the adolescent’s help with household chores. Lastly, the “parental practices that promote family-school connection” encompass indicators of the parents’ direct involvement with school life, such as participating in meetings and following up with school achievements;
- Brazilian Economic Classification Criteria (CCEB, in Portuguese)^(11)^: this system classifies purchasing power into classes A to E, in which A has the highest and E, the lowest purchasing power. The main social classes are segmented according to the purchasing power and householder’s educational level;
- School performance: analyzed with the assessment instruments of the institution where the research took place. Based on the report cards provided by the school, we classified the school achievement into five categories according to the students’ scores (from a total of 100) achieved by the end of the school year. The scores were classified as insufficient (up to 59.99), moderate (60.00 to 69.99), good (70.00 to 79.99), very good (80.00 to 89.99), and excellent (90.00 to 100 points).

The data was collected between June and August 2018 with online Google Forms, which contained the HER, CCEB, and questions to characterize the participating adolescents’ sex, age, and grade in school. The forms were sent to the parents/guardians via e-mail and administered to the students at school during class. The informed consent and assent were signed in paper-based forms.

The response variable of this study was the HER, and the explanatory ones were the sociodemographic aspects (age, sex, and grade in school), CCEB, and school performance. We made a descriptive analysis of the data of the categorical variables with their frequency distribution. The continuous variables were analyzed with measures of central tendency and dispersion. We recategorized the CCEB variables into A and B (instead of B1 and B2) to better analyze them, the householder’s educational level into complete and incomplete high school (including complete middle school/incomplete high school and complete high school/incomplete higher education), and the school performance into excellent, good (instead of very good and good), and moderate.

We used the Mann-Whitney and Kruskal-Wallis tests in the association analyses and set the p-value ≤ 0.05 for the statistically significant associations. We obtained the correlation analysis with the Spearman correlation coefficient, whose magnitude we measured with the following parameter: weak = 0.0 – 0.4; moderate = 0.4 – 0.7; strong = 0.7 – 1.0; the p-value was set at ≤ 0.05^(12)^. We chose these tests because the variables of the HER topics did not have a normal distribution, which was verified with the Shapiro-Wilk test. We entered, processed, and analyzed the data with SPSS, version 21.0.

## RESULTS

The total sample comprised 124 participants – 54.0% females and 46.0% males. Most of them belonged to CCEB class A (66.9%), followed by B1 (25.0%) and B2 (8.1%). Regarding the householder’s educational level, 94.4% had a bachelor’s degree, 4.8% were high school graduates/incomplete higher education, and 0.8% complete middle school/incomplete high school. Most adolescents were 11 years old (27.4%), 24.2% were 12 years old, 25.8% were 13 years old, and 22.6% were 14 years old. Also, 32.3% were in sixth grade, 27.4% were in seventh grade, 21.0% were in eighth grade, and 19.4% were in ninth grade. The school performance of 40.3% of them was classified as very good.

We describe below the descriptive analysis of the raw score in the three HER domains and the analysis of the questions in “resources that promote proximal processes”. The central aspect of this domain, which comprises items 1 to 7, is the parents’ participation in development-stimulating activities, opportunities to interact with the parents, and access to scheduled learning activities (Table 1).

**Table 1:** Descriptive analysis of the raw score in the Home Environment Resources Scale and relative score in the resources that promote proximal processes

	**Variables**	**N**	**Mean**	**SD**	**Median**	**Minimum**	**Q_1_**	**Q_3_**	**Maximum**
**Raw HER score**									
	Out-of-school activities	124	2.97	1.26	3.00	1.00	2.00	4.00	6.00
	Previous year’s outings	124	9.27	3.05	9.00	3.00	7.00	12.00	16.00
	Regular scheduled activities	124	2.38	0.83	2.00	1.00	2.00	3.00	4.00
**Prox. Proc.**	Activities with the parents	124	6.00	1.78	6.00	2.00	5.00	7.00	10.00
	Toys they have or used to have	124	16.23	2.44	17.00	5.00	16.00	18.00	19.00
	Newspapers and magazines available at home	124	1.35	1.12	1.00	0.00	0.00	2.00	4.00
	Books available at home	124	5.44	1.23	6.00	2.00	5.00	6.00	8.00
**F/S**	Schoolwork follow-up	124	11.66	3.88	12.00	0.00	9.00	14.00	18.00
	Routine and schedule	124	12.33	2.37	12.00	6.00	11.00	14.00	16.00
**Stability**	Family time during the week	124	8.76	1.73	9.00	1.00	8.00	10.00	12.00
	**Total score**	124	76.40	10.77	77.50	48.00	69.00	84.00	102.00
**Relative Prox. Proc. score**									
	Out-of-school activities	124	4.58	1.97	5.00	1.43	2.86	5.71	8.57
	Previous year’s outings	124	5.12	1.66	5.00	1.67	3.89	6.58	8.89
	Regular scheduled activities	124	2.88	1.00	2.50	1.11	2.50	3.75	5.00
	Activities with the parents	124	5.96	1.78	6.00	2.00	5.00	7.00	10.00
	Toys they have or used to have	124	8.98	1.36	9.44	2.78	8.89	10.00	10.00
	Newspapers and magazines available at home	124	1.85	1.52	1.43	0.00	0.00	2.86	5.71
	Books available at home	124	7.70	1.66	7.50	2.86	7.14	8.57	10.00

**Caption:** HER = Home Environment Resources Scale; N = number of subjects; SD = standard deviation; Q = quartile; Prox. Proc. = proximal processes; F/S = family-school connection; Stability = Stability of family life

We observed, in the descriptive analysis of school performance, a mean of 80.38, a median of 60.62, and a standard deviation of 8.37.

The association analysis between the HER domains and total score and the sociodemographic data and school performance, with Mann-Whitney and Kruskal-Wallis tests, revealed a statistically significant association between: “resources that promote proximal processes” and age (p = 0.002) and grade in school (p = 0.013); “parental practices that promote family-school connection” and age (p = 0.001) and grade in school (p = 0.003); total HER score and age (p = 0.001) and grade in school (p = 0.005). The other associations were not statistically significant (Table 2).

**Table 2:** Association analysis of the domains in the Home Environment Resources Scale and its total score with sociodemographic data and school performance

**Variables**	Sex		**Age (years)**				**Grade**				**Householder educational level**		**CCEB**		**School performance**		
	**Fem.**	**Mal.**	**11**	**12**	**13**	**14**	**6^th^**	**7^th^**	**8^th^**	**9^th^**	**Up to I.H.E.**	**B.D.**	**A**	**B**	**Exc.**	**Good**	**Mod.**
**Proximal**																	
**Processes**																	
Mean	44.12	43.11	47.26	42.10	43.53	41.07	46.13	42.41	44.23	40.67	38.67	43.96	44.02	42.90	45.07	43.92	41.11
Median	45.00	43.00	47.00	43.00	44.00	42.00	47.00	44.00	44.00	40.50	39.50	45.00	45.00	44.00	45.00	45.00	43.00
Standard deviation	6.96	7.76	5.47	7.91	8.05	6.34	6.40	7.64	7.81	6.70	12.18	6.99	7.29	7.43	4.46	7.31	8.94
p-value	0.473^1^	0.002^2^*	0.013^2^*	0.325^1^	0.340^1^	0.413^2^
**Family-School**																	
Mean	11.36	12.02	13.26	12.26	11.31	9.50	13.23	11.71	10.81	9.92	9.83	11.75	11.43	12.12	12.20	11.57	11.67
Median	12.00	13.00	14.00	13.00	12.00	9.50	14.00	12.00	12.00	11.50	11.00	12.00	12.00	13.00	12.00	12.00	12.00
Standard deviation	4.00	3.74	3.72	3.12	4.25	3.48	3.57	3.51	4.33	3.57	6.01	3.78	4.07	3.48	3.36	4.00	3.84
p-value	0.269^1^		0.001^2^*				0.003^2^^*^				0.800^1^		0.447^1^		0.959^2^		
**Stability**																	
Mean	20.88	21.33	21.65	21.20	20.69	20.75	21.78	20.56	21.15	20.63	18.67	21.22	21.04	21.20	22.07	21.18	19.83
Median	21.00	22.00	22.00	22.00	21.00	21.00	22.00	22.00	21.50	20.50	19.50	22.00	22.00	21.00	22.00	22.00	21.00
Standard deviation	3.67	2.98	3.00	3.80	3.53	3.17	3.12	4.19	2.68	3.12	2.81	3.37	3.36	3.42	3.08	3.21	4.13
p-value	0.595^1^		0.661^2^				0.481^2^				0.141^1^		0.833^1^		0.273^2^		
**Total HER**																	
Mean	76.36	76.46	82.18	75.53	75.53	71.32	81.13	74.68	76.19	71.21	67.17	76.93	76.46	76.22	79.33	76.67	72.61
Median	78.00	77.00	81.00	77.50	74.50	71.50	80.00	77.00	74.50	71.50	66.00	78.00	77.00	79.00	78.00	78.00	75.00
Standard deviation	10.34	11.33	8.90	10.56	11.35	9.65	9.02	11.28	10.66	10.23	13.21	10.53	10.77	10.89	7.24	10.79	12.43
p-value	0.982^1^		0.001^2^ *				0.005^2*^				0.116^1^		0.782^1^		0.252^2^		

1 Mann-Whitney test;

2 Kruskal-Wallis test

* = p ≤ 0.05

**Caption:** Fem. = Females; Mal. = Males; fam. = family; CCEB = Brazilian Economic Classification Criteria; I.H.E. = Incomplete higher education; B.D. = bachelor’s degree; Exc. = excellent; Mod. = moderate; HER = Home Environment Resources Scale

The correlation analysis revealed statistical significance between the scores in “resources that promote proximal processes”. The following had weak positive correlations: out-of-school activities with previous year’s outings (0.254), activities with the parents (0.391), toys (0.201), newspapers and magazines available at home (0.178), and books available at home (0.267); previous year’s outings with regular scheduled activities (0.205) and books available at home (0.279); regular scheduled activities with the activities with the parents (0.200); activities with the parents with toys and books available at home (0.288 and 0.228, respectively); toys with newspapers and magazines available at home (0.214) and books available at home (0.232). There was a moderate positive correlation between activities with the parents (0.442) and toys (0.413). All correlations were statistically significant (Table 3).

**Table 3:** Correlation between relative scores in the proximal aspects of the Home Environment Resources Scale

**Variables**	**Out-of-school activities**	**Previous year’s outings**	**Regular scheduled activities**	**Activities with the parents**	**Toys they have or used to have**	**Newspapers and magazines available at home**	**Books available at home**
**Out-of-school activities**	1.000	0.254*	0.172	0.391*	0.201*	0.178*	0.267*
**Previous year’s outings**		1.000	0.205*	0.442*	0.413*	0.170	0.279*
**Regular scheduled activities**			1.000	0.200*	0.073	0.064	0.141
**Activities with the parents**				1.000	0.288*	0.031	0.228*
**Toys they have or used to have**					1.000	0.214*	0.232*
**Newspapers and magazines available at home**						1.000	0.002
**Books available at home**							1.000

Spearman coefficient

* = significant correlation (p ≤ 0.05)

We made a correlation analysis between raw scores in “resources that promote proximal processes”. The following had weak positive correlations: out-of-school activities with previous year’s outings (0.239), regular scheduled activities (0.183), activities with the parents (0.382), toys (0.208), newspapers and magazines available at home (0.226), and books available at home (0.287); previous year’s outings with regular scheduled activities, newspapers and magazines available at home, and books available at home (0.215, 0.188, and 0.290, respectively); regular scheduled activities with activities with the parents (0.209); activities with the parents with toys (0.282) and books available at home (0.222); toys with newspapers and magazines available at home and books available at home (0.223 and 0.243, respectively). There was a moderate correlation between the previous year’s outings and activities with the parents (0.437) and toys (0.416). All correlations were statistically significant (Table 4).

**Table 4:** Correlation between raw scores in the proximal aspects of the Home Environment Resources Scale

**Variables**	**Out-of-school activities**	**Previous year’s outings**	**Regular scheduled activities**	**Activities with the parents**	**Toys they have or used to have**	**Newspapers and magazines available at home**	**Books available at home**
**Out-of-school activities**	1.000	0.239*	0.183*	0.382*	0.208*	0.226*	0.287*
**Previous year’s outings**		1.000	0.215*	0.437*	0.416*	0.188*	0.290*
**Regular scheduled activities**			1.000	0.209*	0.102	0.092	0.148
**Activities with the parents**				1.000	0.282*	0.042	0.222*
**Toys they have or used to have**					1.000	0.223*	0.243*
**Newspapers and magazines available at home**						1.000	0.001
**Books available at home**							1.000

Spearman coefficient

* = significant correlation (p ≤ 0.05)

The correlation analysis between raw HER scores per domain and total and mean school performance revealed a strong positive correlation between total HER score and “resources that promote proximal processes” (0.811) and “parental practices that promote family-school connection” (0.708); a moderate positive correlation between total HER score and “predictable activities that indicate some degree of stability of family life” (0.554); and a weak correlation between “parental practices that promote family-school connection” and “resources that promote proximal processes” (0.355) and “predictable activities that indicate some degree of stability of family life” (0.325) (Table 5).

**Table 5:** Correlation between the scores in the Home Environment Resources Scale per domain and the mean school performance

**Variables**	**Proximal processes**	**Family-school connection**	**Activities that indicate stability**	**Total HER**	**Mean S.P.**
**Proximal processes**	1.000	0.355*	0.140	0.811*	0.158
**Family-school connection**		1.000	0.325*	0.708*	0.004
**Activities that indicate stability**			1.000	0.554*	0.533
**Total HER**				1.000	0.121
**Mean S.P.**					1.000

Spearman coefficient

* = significant correlation (p ≤ 0.05)

**Caption** HER = Home Environment Resources Scale; S.P. = school performance

We made a correlation analysis between the HER questions and the mean school performance. There was a weak positive correlation between the seventh question (whether they have books available at home) and the participants’ mean school performance.

## DISCUSSION

This study investigated the relationship between home resources, sociodemographic aspects, and school performance of middle school students. We used the HER, CCEB, and the participants’ school performance and sociodemographic data – age, sex, grade in school, and parents’ educational level.

The study sample had a balanced distribution regarding sex, grade in school, age, and school performance. It is important to highlight that most of them belonged to social class A and reported that their householder had a bachelor’s degree – which does not portray the national scenario, although it corroborates data on the social classes with the highest incidence in this research^(9)^.

The parents’ school records, including flunks and years at school, influence their children’s school performance^(9,13-15)^. The higher the parents’ educational level, the higher the chances of better access to higher social strata. This influences the availability of resources and, consequently, the adolescent’s school performance, frequency and type of leisure activities, and nonschool activities^(9)^. This process creates a cycle in which adolescents whose families have a better socioeconomic condition and educational level have more resources. Also, their parents’ condition favors their participation in the children’s school life. In contrast, the income of families in social vulnerability is spent on basic needs, with little money available for leisure^(9)^.

We verified that only newspapers and magazines available at home were not reported. Therefore, the availability of environment resources in classes A and B can be questioned at first. The literature indicates that socioeconomic conditions affect the distribution of resources, as demonstrated in access to outings, toys, and books – which are associated with greater comfort, resulting from home amenities^(16)^. However, the availability of newspapers and magazines, as portrayed in HER, is no longer compatible with present-day information consumption. Accordingly, few parents reported having printed newspapers and magazines at home, which highlights the ongoing migration to digital news consumption since the time the instrument was developed. According to data from the *Instituto Verificador de Comunicação* (ICV – Media Verification Institute)^(17)^, the circulation of digital newspapers and magazines has increased, while that of printed ones has dropped.

Given that most of this study sample had either an excellent or good school performance – although the sample had been randomized –, we can infer some possible reasons for such results, namely: the parents that agreed to participate in the research were the most interested ones, who better follow up with their children; or the school profile and its follow-up method; or yet classes A and B’s greater access to resources, which can positively influence school performance and impact the results. It has been observed that people in social vulnerability have fewer resources available and the family context can cause maladjustments and negatively interfere with school performance^(1)^.

The associations found between the parents’ participation in stimulating experiences, the age, and grade in school; between “parental practices that promote family-school connection”, the age, and grade in school; and between total HER score, the age, and grade in school evidence changes in the adolescents’ interaction with their parents and with tasks throughout their school life. The weak positive correlations between the previous year’s outings, the age, and grade in school corroborate a previous study, which found that the relationship between family, school, and the resources provided by the parents, as well as how they used them, changes as they grow older^(18)^.

Out-of-school activities had a weak positive correlation with other HER topics, evidencing the importance of two elements: parental presence and resource availability. This relationship was also evident in the interaction between regular scheduled activities, books available at home, and the previous year’s outings. These results may indicate that outings represent a part of the family activities.

The previous year’s outings also had a weak positive correlation with regular scheduled activities and books, magazines, and newspapers available at home. The moderate positive correlation between toys and activities with the parents, and the weak positive correlation between activities with the parents and regular scheduled activities, toys, and books may be due not only to these objects’ availability but also to the interaction between parents and children^(9,19)^. Research indicates that having books and educational toys available and counting with parental schoolwork follow-up are factors related to better school performance^(9)^.

The importance of interacting with the parents, having toys and books available, enjoying spare time well, and having access to scheduled learning activities and material to stimulate reasoning becomes evident when we analyze their influence on the raw scores of the other domains. Students with a satisfactory school performance interact more often with their parents, have books available at home, have leisure activities, and keep a regular routine^(19)^.

Furthermore, the study revealed a weak positive correlation between book availability and the participants’ mean school performance. These data reinforce the idea that having more resources at home – with emphasis on books available to adolescents – has a positive influence on school performance^(9,19)^.

The analysis between school performance and the total score in HER and in each of its three domains (proximal processes, family-school connection, and parental practices that indicate some degree of stability) revealed important correlations. We found a strong positive correlation between total HER score and “resources that promote proximal processes” and “parental practices that promote family-school connection”. These results evidence the importance of parental presence and participation to their children’s school life. The “predictable activities that indicate some degree of stability of family life” also revealed a positive, though moderate correlation with total HER score.

Also, “parental practices that promote family-school connection” had a weak positive correlation with “resources that promote proximal processes” and “predictable activities that indicate some degree of stability of family life”. We must highlight that a healthy family-school relationship is significant and beneficial to the educational process^(9)^ because it can influence the resources that promote proximal processes^(20,21)^.

The strong positive correlation between total HER score, “resources that promote proximal processes”, and “parental practices that promote family-school connection”; and the moderate positive correlation between the “predictable activities that indicate some degree of stability of family life”, total HER score, and school performance reinforce that having a routine, activities with the parents and books are elements that reflect on the student’s school performance^(16)^. Book availability also seems to influence grades, as it had a weak positive correlation with school performance. There is evidence that parental practices can influence infrequent word reading performance and that parent-child communication broadens knowledge^(22)^.

The findings of the investigation reported in this text point out that social class, the parents’ educational level, and home resources – particularly book availability – are factors that influence school performance. Moreover, we can infer that parental participation in stimulating experiences, establishing routines, and maintaining a good family-school relationship is also essential to good academic results.

It is important to be cautious when analyzing the results of this study, as they are related only to the reference population – i.e., middle school adolescents from a specific context. Therefore, they cannot be generalized. Furthermore, there is no direct relationship between poor performance and lack of resources, as the students’ grades may be influenced by other variables, such as the institution’s assessment method and teaching approach. Thus, further studies are needed to investigate the relationship between family resources and school performance in different scenarios and better clarify how these associations and relationships take place.

This research pointed out advances in the relationships between family resources, sociodemographic factors, parents’ educational level, and performance in a different context from what is usually found in the literature. It also showed a correlation between the previous year’s outings and activities with the parents and toys.

## CONCLUSION

The data analysis showed that age and grade in school are associated with “parental practices that promote family-school connection” and “resources that promote proximal processes”. We also analyzed the correlations between family resources, sociodemographic data, and the participants’ school performance. The research revealed the importance of a positive family-school relationship, family outings, access to toys and books available at home to the adolescents’ school performance.
