# Supplementary material for: Family resources and school performance: an analysis of associated factors in middle school adolescents
Source: Codas. 2022 Jan 5;34(2):e20210058. doi: 10.1590/2317-1782/20212021058 (PMC9851186; doi:10.1590/2317-1782/20212021058)
Supplement: Translation

# Translation: Recursos do ambiente familiar e desempenho escolar: análise de fatores associados em adolescentes do ensino fundamental

**Authors:** Rebeca Mota Cabral e Silva, Graziela Nunes Alfenas Fernandes, Andrezza Gonzalez Escarce, Stela Maris Aguiar Lemos

**Affiliations:** 1 Departamento de Fonoaudiologia, Faculdade de Medicina, Universidade Federal de Minas Gerais – UFMG - Belo Horizonte (MG), Brasil.

**Keywords:** Adolescente, Desempenho Acadêmico, Família, Classe Social, Fonoaudiologia

## Abstract

**Objetivo:**

Verificar a associação entre recursos do ambiente familiar, aspectos sociodemográficos e desempenho escolar de estudantes do Ensino Fundamental.

**Método:**

Estudo observacional analítico transversal, com amostra probabilística estratificada por sexo, idade e ano escolar. Participaram 124 adolescentes, de ambos os sexos, entre 11 e 14 anos, matriculados no Ensino Fundamental II de uma instituição particular. Os responsáveis receberam um formulário online contendo: Inventário dos Recursos do Ambiente Familiar (RAF), Critério de Classificação Econômica Brasil (CCEB) e questões sociodemográficas. O desempenho escolar foi fornecido pela instituição. Realizou-se distribuição de frequência das variáveis categóricas e medidas de tendência central e de dispersão para as contínuas. Utilizou-se os testes Mann-Whitney, Kruskal-Wallis e coeficiente de correlação de Spearman, sendo considerados significantes os que apresentaram valor de p≤0,05.

**Resultados:**

A maioria da amostra possuía 11 anos, do sexo feminino, cursava o 6º ano do Ensino Fundamental, obteve desempenho escolar muito bom, apresentava classificação A e declarou que a/o chefe de família possuía Ensino Superior completo. Houve associação entre idade e ano escolar com a interação com os pais, ligação família-escola e RAF total. Os processos proximais demonstraram correlação positiva de magnitude moderada com passeios e brinquedos. Houve correlação positiva moderada entre total do RAF e atividades previsíveis que sinalizam estabilidade. Os processos proximais se correlacionaram de maneira positiva forte com a ligação família-escola. Ter livros em casa revelou correlação positiva fraca com o desempenho escolar.

**Conclusão:**

Os recursos do ambiente familiar apresentaram relação com o desempenho escolar dos adolescentes.

## INTRODUÇÃO

O desempenho escolar é reflexo de uma série de elementos. Sob a concepção de que o mau desempenho escolar não é apenas resultado do que o adolescente realiza na escola, pode-se considerar que o sucesso acadêmico é determinado por múltiplas variáveis. Fatores ligados ao estudante e aos seus diversos contextos – familiar, social, cultural, institucional, político e econômico – têm sido apontados como agentes influenciadores dos resultados dos escolares^(1)^. O ambiente pode influenciar de forma negativa ou positiva no desenvolvimento de um indivíduo, e a família é apontada como contexto fundamental no processo de aprendizagem de crianças e adolescentes^(2,3)^. Estudos indicam que o envolvimento dos pais nas atividades escolares de seus filhos é um dos elementos que podem repercutir no interesse destes pelas atividades escolares, além de evidenciar a importância que estes dão à educação^(4-6)^.

A interação entre a família e a escola atrai a atenção dos pesquisadores^(4,7)^, uma vez que o envolvimento dos pais na educação tem sido apontado como elemento fundamental para um bom desempenho acadêmico^(7)^. Os fenômenos ligados à educação e à relação entre família e escola representam uma parcela expressiva das produções de professores, de gestores, de pedagogos e de especialistas em educação que se preocupam com essa relação. Este interesse deriva da necessidade de responder a questionamentos importantes sobre a profundidade dos efeitos desta interação, sobre desvendar os possíveis benefícios ou malefícios e o real papel do vínculo entre a família e a escola na formação integral de um estudante^(7)^. Com o objetivo de conhecer o estado da arte do tema “Relação Família-Escola” no Brasil, uma revisão sistemática selecionou estudos que demonstraram que a boa relação entre a escola e pais pode, inclusive, prevenir comportamentos disfuncionais^(8)^.

As experiências vividas na fase escolar podem interferir na trajetória e no futuro do indivíduo, e o fracasso escolar aparece como um adjuvante no aumento da vulnerabilidade e da diminuição da autoestima^(9)^. Compreender fatores que influenciam o baixo nível de desempenho nos diferentes contextos pode auxiliar no desenvolvimento de estratégias de combate ao mau desempenho escolar.

O objetivo do presente estudo foi verificar a associação entre recursos do ambiente familiar com base nos dados obtidos por meio do Inventário dos Recursos do Ambiente Familiar, aspectos sociodemográficos e desempenho escolar de estudantes do Ensino Fundamental.

## MÉTODO

Trata-se de estudo observacional, analítico e transversal, com amostra probabilística estratificada por sexo, idade e ano escolar. Participaram da pesquisa adolescentes de ambos os sexos, de 11 a 14 anos, matriculados no Ensino Fundamental II de uma instituição integrante da rede particular de ensino do município de Belo Horizonte/MG. Os pais ou responsáveis e os adolescentes que aceitaram participar assinaram, respectivamente, o Termo de Consentimento Livre e Esclarecido e o Termo de Assentimento Livre e Esclarecido.

Este estudo cumpriu as diretrizes e normas regulamentadoras de pesquisa envolvendo seres humanos estabelecidas na Resolução 466 de 12 de dezembro de 2012 e foi aprovado pelo Comitê de Ética em Pesquisa da instituição sob o número do parecer 2.422.795.

Como critérios de inclusão na pesquisa era necessário ter entre 11 e 14 anos e estar regularmente matriculado no Ensino Fundamental da instituição pesquisada. Foram excluídos deste estudo indivíduos com alterações cognitivas, neurológicas ou psiquiátricas que impedissem a realização da pesquisa, condição observada no comportamento durante a aplicação dos questionários.

Para cumprir o objetivo da pesquisa foram realizados os seguintes procedimentos:

- O Inventário de Recursos do Ambiente Familiar (RAF)^(10)^: questionário que avalia os aspectos do ambiente familiar que podem interferir no desempenho escolar. É composto por dez perguntas de múltipla escolha categorizadas em três amplos domínios: *recursos que promovem processos proximais*; *atividades previsíveis que sinalizam algum grau de estabilidade na vida familiar*; e *práticas parentais que promovem a ligação família-escola*. Os *recursos que promovem processos proximais* abrangem áreas e atividades como a participação dos pais em experiências estimuladoras, oportunidades de interação com os pais e acesso a atividades programadas de aprendizagem. As *atividades previsíveis que sinalizam algum grau de estabilidade na vida familiar* incluem rotinas e reuniões regulares da família e cooperação do adolescente em tarefas domésticas. Por fim, as *práticas parentais que promovem a ligação família-escola* englobam indicadores de envolvimento direto dos pais na vida escolar, como participação nas reuniões e acompanhamento do rendimento escolar;
- Critério de Classificação Econômica Brasil (CCEB)^(11)^: consiste em um sistema que define o poder aquisitivo em classes de A a E – sendo a classe A com maior poder aquisitivo e a classe E, com menor poder aquisitivo. As grandes classes econômicas são segmentadas pelo poder aquisitivo e pelo grau de instrução do chefe da família;
- Desempenho escolar: a análise do desempenho escolar foi realizada de acordo com os instrumentos de avaliação da instituição pesquisada. Com base no boletim fornecido pela escola, o resultado do aproveitamento escolar foi apresentado em cinco categorias que classificam o desempenho escolar de acordo com a quantidade de pontos – de um total de 100 – conquistados pelo aluno ao final do ano letivo. As pontuações foram classificadas como insuficiente até 59,99; regular de 60,00 a 69,99; bom de 70,00 a 79,99; muito bom de 80,00 a 89,99; e excelente de 90,00 a 100 pontos;

A coleta dos dados foi realizada entre junho e agosto de 2018 por meio de formulários online criados no Google Forms, o qual continha: o Inventário de Recursos do ambiente familiar (RAF) e o Critério de Classificação Econômica Brasil (CCEB), além de perguntas de caracterização dos participantes relativas ao sexo, à idade e ao ano escolar do adolescente. Os formulários foram enviados por correio eletrônico aos responsáveis e aplicados aos estudantes no ambiente e horário escolares. Os termos de consentimento e assentimento livres e esclarecidos foram preenchidos e assinados na forma física.

O Inventário de Recursos do Ambiente Familiar (RAF) foi considerado a variável resposta deste estudo. Foram consideradas variáveis explicativas os aspectos sociodemográficos (idade, sexo e ano escolar), a CCEB e o desempenho escolar. Para as variáveis categóricas foi realizada uma análise descritiva dos dados, por meio da distribuição de frequência das variáveis categóricas. As variáveis contínuas foram analisadas por meio de medidas de tendência central e de dispersão. As variáveis do CCEB foram recategorizadas em A e B (antes B1 e B2) para melhor análise. O grau de instrução do chefe de família foi recategorizado em Ensino Superior completo e incompleto (até antigos Ensino Fundamental II completo/Médio incompleto e Médio completo/Superior incompleto) e o desempenho escolar foi recategorizado em excelente, bom (antes muito bom e bom) e regular.

Foram utilizados os testes Mann-Whitney e Kruskal-Wallis para as análises de associação, e foram consideradas como associações estatisticamente significativas as que apresentaram valor de p ≤ 0,05. A análise de correlação foi obtida pelo coeficiente de correlação de Spearman, com o qual a magnitude da correlação foi medida seguindo o seguinte parâmetro: fraca = 0,0-0,4; moderada = 0,4-0,7 e forte = 0,7-1,0; com valor de p ≤ 0,05^(12)^. A escolha dos testes deve-se ao fato de que as variáveis dos tópicos do Inventário do RAF não apresentaram distribuição normal, o que foi verificado por meio do teste Shapiro-Wilk. Para entrada, processamento e análise dos dados foi utilizado o *software* SPSS versão 21.0.

## RESULTADOS

A amostra total foi composta por 124 participantes, dos quais 54,0% pertenciam ao sexo feminino e 46,0% pertenciam ao sexo masculino. Verificou-se que a maioria apresentou classificação A no CCEB (66,9%), seguida pela B1 (25,0%), e, por último B2 (8,1%). Em relação ao nível de instrução do chefe de família, foi observado que 94,4% possuíam Ensino Superior completo, 4,8% possuíam Ensino Médio completo/superior incompleto, e 0,8% Fundamental II completo/Médio incompleto. Foi verificado que a maior parte dos adolescentes possuía 11 anos (27,4%), sendo que 24,2% possuíam 12 anos, 25,8% possuíam 13 anos e 22,6% possuíam 14 anos. Foi observado que 32,3% cursavam o 6º ano do Ensino Fundamental, 27,4% cursavam o 7º ano, 21,0% cursavam o 8º ano e 19,4% cursavam o 9º ano. Da amostra total, 40,3% obtiveram desempenho muito bom na classificação do desempenho escolar.

A análise descritiva da pontuação bruta dos três domínios do RAF e a análise relativa às perguntas pertencentes ao domínio *recursos que promovem processos proximais* estão descritas a seguir. Este domínio é composto pelos itens de 1 a 7 e tem como temática central a participação dos pais em atividades estimuladoras do desenvolvimento, as oportunidades de interação com os pais e o acesso às atividades programadas de aprendizagem (Tabela 1).

**Tabela 1:** Análise descritiva da pontuação bruta do Inventário dos Recursos do Ambiente Familiar e da pontuação relativa dos recursos que promovem processos proximais.

	**Variáveis**	**N**	**Média**	**D.P.**	**Mediana**	**Mínimo**	**1º Q**	**3º Q**	**Máximo**
**Pontuação bruta do RAF**									
	Atividades fora da escola	124	2,97	1,26	3,00	1,00	2,00	4,00	6,00
	Passeios do último ano	124	9,27	3,05	9,00	3,00	7,00	12,00	16,00
	Atividades regulares programadas	124	2,38	0,83	2,00	1,00	2,00	3,00	4,00
**Proc. Prox.**	Atividades com os pais	124	6,00	1,78	6,00	2,00	5,00	7,00	10,00
	Brinquedos que tem ou teve	124	16,23	2,44	17,00	5,00	16,00	18,00	19,00
	Possui jornais e revistas em casa	124	1,35	1,12	1,00	0,00	0,00	2,00	4,00
	Possui livros em casa	124	5,44	1,23	6,00	2,00	5,00	6,00	8,00
**F/E**	Acompanhamento nos afazeres escolares	124	11,66	3,88	12,00	0,00	9,00	14,00	18,00
	Rotina e horários	124	12,33	2,37	12,00	6,00	11,00	14,00	16,00
**Estabilidade**	Convivência familiar durante a semana	124	8,76	1,73	9,00	1,00	8,00	10,00	12,00
	**Pontuação total**	124	76,40	10,77	77,50	48,00	69,00	84,00	102,00
**Pontuação relativa dos Proc. Prox.**									
	Atividades fora da escola	124	4,58	1,97	5,00	1,43	2,86	5,71	8,57
	Passeios do último ano	124	5,12	1,66	5,00	1,67	3,89	6,58	8,89
	Atividades regulares programadas	124	2,88	1,00	2,50	1,11	2,50	3,75	5,00
	Atividades com os pais	124	5,96	1,78	6,00	2,00	5,00	7,00	10,00
	Brinquedos que tem ou teve	124	8,98	1,36	9,44	2,78	8,89	10,00	10,00
	Possui jornais e revistas em casa	124	1,85	1,52	1,43	0,00	0,00	2,86	5,71
	Possui livros em casa	124	7,70	1,66	7,50	2,86	7,14	8,57	10,00

**Legenda:** RAF= Inventário dos Recursos do Ambiente Familiar; N= número de indivíduos; D.P.= desvio padrão; Q= quartil; Proc.Prox= Processos Proximais; F/E= Ligação Família-Escola; Estabilidade= Estabilidade na vida familiar

Na análise descritiva da variável desempenho escolar foram observadas: média 80,38; mediana 60,62 e desvio padrão 8,37.

A análise de associação entre os domínios e a pontuação total do RAF com os dados sociodemográficos e o desempenho escolar, por meio dos testes Mann-Whitney e Kruskal-Wallis, revelou que houve associação com significância estatística entre: *recursos que promovem processos proximais* com a idade (p=0,002) e com o ano escolar (p=0,013); *práticas parentais que promovem a ligação família-escola* com a idade (p=0,001) e com o ano escolar (p=0,003); RAF total com a idade (p=0,001) e com o ano escolar (p=0,005). As demais associações não revelaram resultados com significância estatística (Tabela 2).

**Tabela 2:** Análise de associação dos domínios do Inventário dos Recursos do Ambiente Familiar e a pontuação total com os dados sociodemográficos e desempenho escolar

**Variáveis**	**Sexo**		**Idade (anos)**				**Ano escolar**				**Instrução chefe da família**		**CCEB**		**Desempenho Escolar**		
	**Fem.**	**Masc.**	**11**	**12**	**13**	**14**	**6º**	**7º**	**8º**	**9º**	**Até S.I.**	**S.C.**	**A**	**B**	**Exc.**	**Bom**	**Reg.**
**Processos**																	
**Proximais**																	
Média	44,12	43,11	47,26	42,10	43,53	41,07	46,13	42,41	44,23	40,67	38,67	43,96	44,02	42,90	45,07	43,92	41,11
Mediana	45,00	43,00	47,00	43,00	44,00	42,00	47,00	44,00	44,00	40,50	39,50	45,00	45,00	44,00	45,00	45,00	43,00
Desvio padrão	6,96	7,76	5,47	7,91	8,05	6,34	6,40	7,64	7,81	6,70	12,18	6,99	7,29	7,43	4,46	7,31	8,94
p-valor	0,473^1^		0,002^2^*				0,013^2^*				0,325^1^		0,340^1^		0,413^2^		
**Família-escola**																	
Média	11,36	12,02	13,26	12,26	11,31	9,50	13,23	11,71	10,81	9,92	9,83	11,75	11,43	12,12	12,20	11,57	11,67
Mediana	12,00	13,00	14,00	13,00	12,00	9,50	14,00	12,00	12,00	11,50	11,00	12,00	12,00	13,00	12,00	12,00	12,00
Desvio padrão	4,00	3,74	3,72	3,12	4,25	3,48	3,57	3,51	4,33	3,57	6,01	3,78	4,07	3,48	3,36	4,00	3,84
p-valor	0,269^1^		0,001^2^*				0,003^2^*				0,800^1^		0,447^1^		0,959^2^		
**Estabilidade**																	
Média	20,88	21,33	21,65	21,20	20,69	20,75	21,78	20,56	21,15	20,63	18,67	21,22	21,04	21,20	22,07	21,18	19,83
Mediana	21,00	22,00	22,00	22,00	21,00	21,00	22,00	22,00	21,50	20,50	19,50	22,00	22,00	21,00	22,00	22,00	21,00
Desvio padrão	3,67	2,98	3,00	3,80	3,53	3,17	3,12	4,19	2,68	3,12	2,81	3,37	3,36	3,42	3,08	3,21	4,13
p-valor	0,595^1^		0,661^2^				0,481^2^				0,141^1^		0,833^1^		0,273^2^		
**RAF Total**																	
Média	76,36	76,46	82,18	75,53	75,53	71,32	81,13	74,68	76,19	71,21	67,17	76,93	76,46	76,22	79,33	76,67	72,61
Mediana	78,00	77,00	81,00	77,50	74,50	71,50	80,00	77,00	74,50	71,50	66,00	78,00	77,00	79,00	78,00	78,00	75,00
Desvio padrão	10,34	11,33	8,90	10,56	11,35	9,65	9,02	11,28	10,66	10,23	13,21	10,53	10,77	10,89	7,24	10,79	12,43

p-valor

0,982^1^

0,001^2*^

0,005^2*^

0,116^1^

0,782^1^

0,252^2^

1 Teste Mann-Whitney;

2 Teste Kruskal-Wallis

* = p≤0,05

**Legenda:** Fem.= Feminino; Masc.= Masculino; fam.= família; CCEB= Critério de Classificação Econômica Brasil; S.I.= Superior Incompleto; S.C.= Superior Completo; Exc.= Excelente; Reg.= Regular; RAF= Inventário dos Recursos do Ambiente Familiar

A análise de correlação evidenciou significância estatística entre as pontuações relativas dos itens pertencentes ao domínio dos *recursos que promovem processos proximais*. Foi possível observar correlação positiva de magnitude fraca entre: as atividades realizadas fora da escola com os passeios do último ano (0,254), com as atividades com os pais (0,391), com os brinquedos (0,201), com o acesso a jornais e revistas em casa (0,178) e com a existência de livros em casa (0,267); os passeios realizados no último ano com as atividades regulares programadas (0,205) e com a existência livros em casa (0,279); as atividades regulares programadas com as atividades realizadas com os pais (0,200); as atividades realizadas com os pais com os brinquedos e com a existência de livros em casa (0,288 e 0,228, respectivamente); os brinquedos com o acesso a jornais e revistas em casa (0,214) e com a existência de livros em casa (0,232). Houve correlação positiva de magnitude moderada entre as atividades com os pais (0,442) e os brinquedos (0,413). Todas as correlações citadas apresentaram significância estatística (Tabela 3).

**Tabela 3:** Correlação entre a pontuação relativa dos aspectos proximais do Inventário dos Recursos do Ambiente Familiar

**Variáveis**	**AtivAtividades fora da escola**	**Passeios do último ano**	**Atividades regulares programadas**	**Atividades com os pais**	**Brinquedos que tem ou teve**	**Possui jornais e revistas em casa**	**Possui livros em casa**
**Atividades fora da escola**	1,000	0,254*	0,172	0,391*	0,201*	0,178*	0,267*
**Passeios do último ano**		1,000	0,205*	0,442*	0,413*	0,170	0,279*
**Atividades regulares programadas**			1,000	0,200*	0,073	0,064	0,141
**Atividades com os pais**				1,000	0,288*	0,031	0,228*
**Brinquedos que tem ou teve**					1,000	0,214*	0,232*
**Possui jornais e revistas em casa**						1,000	0,002
**Possui livros em casa**							1,000

Coeficiente de Spearman

* = correlação significante (p≤0,05)

Realizou-se análise de correlação entre as pontuações brutas dos itens pertencentes ao domínio *recursos que promovem processos proximais*, na qual foi observada correlação positiva de magnitude fraca entre: as atividades fora da escola com os passeios realizados no último ano (0,239), com as atividades regulares programadas (0,183), com as atividades com os pais (0,382), com os brinquedos (0,208), com o acesso a jornais e revistas em casa (0,226) e com a existência de livros em casa (0,287); os passeios do último ano com as atividades regulares programadas, com o acesso a jornais e revistas em casa e com a existência de livros em casa (0,215; 0,188 e 0,290, respectivamente); as atividades regulares programadas com as atividades com os pais (0,209); as atividades com os pais com os brinquedos (0,282) e com a existência de livros em casa (0,222); os brinquedos com o acesso a jornais e revistas em casa e com a existência livros em casa (0,223 e 0,243, respectivamente). Foi encontrada a existência de correlação de magnitude moderada entre passeios do último ano, as atividades com os pais (0,437) e brinquedos (0,416). Todas as correlações citadas apresentaram significância estatística (Tabela 4).

**Tabela 4:** Correlação entre a pontuação bruta dos aspectos proximais do Inventário dos Recursos do Ambiente Familiar

**Variáveis**	**Atividades fora da escola**	**Passeios do último ano**	**Atividades regulares programadas**	**Atividades com os pais**	**Brinquedos que tem ou teve**	**Possui jornais e revistas em casa**	**Possui livros em casa**
**Atividades fora da escola**	1,000	0,239*	0,183*	0,382*	0,208*	0,226*	0,287*
**Passeios do último ano**		1,000	0,215*	0,437*	0,416*	0,188*	0,290*
**Atividades regulares programadas**			1,000	0,209*	0,102	0,092	0,148
**Atividades com os pais**				1,000	0,282*	0,042	0,222*
**Brinquedos que tem ou teve**					1,000	0,223*	0,243*
**Possui jornais e revistas em casa**						1,000	0,001
**Possui livros em casa**							1,000

Coeficiente de Spearman

* = correlação significante (p≤0,05)

A análise de correlação entre as pontuações brutas do RAF por domínio, o total e a média do desempenho escolar evidenciou correlação positiva de magnitude forte entre o RAF total com os domínios recursos que promovem processos proximais (0,811) e práticas parentais que promovem a ligação família-escola (0,708); correlação positiva de magnitude moderada entre RAF total e as atividades previsíveis que sinalizam algum grau de estabilidade na vida familiar (0,554) e de magnitude fraca entre as práticas parentais que promovem a ligação família-escola com os recursos que promovem processos proximais (0,355) e com as atividades previsíveis que sinalizam algum grau de estabilidade na vida familiar (0,325) (Tabela 5).

**Tabela 5:** Correlação entre a pontuação do Inventário dos Recursos do Ambiente Familiar por domínios e a média do desempenho escolar

**Variáveis**	**Processos proximais**	**Ligação Família Escola**	**Atividades que sinalizam estabilidade**	**RAF total**	**Média D.E.**
**Processos Proximais**	1,000	0,355*	0,140	0,811*	0,158
**Ligação Família-Escola**		1,000	0,325*	0,708*	0,004
**Atividades que sinalizam estabilidade**			1,000	0,554*	0,533
**RAF total**				1,000	0,121
**Média D.E.**					1,000

Coeficiente de Spearman

* = correlação significante (p≤0,05)

**Legenda:** RAF= Inventário dos Recursos do Ambiente Familiar; D.E.= Desempenho Escolar

Foi realizada análise de correlação entre as questões do Inventário RAF e a média do desempenho escolar, a qual revelou a presença de correlação positiva de magnitude fraca entre a sétima pergunta, que questiona a existência de livros disponíveis na casa, e o desempenho escolar médio dos participantes.

## DISCUSSÃO

Este estudo investigou a relação entre os recursos do ambiente familiar, os aspectos sociodemográficos e o desempenho escolar de estudantes do Ensino Fundamental II. Foram utilizados como instrumentos o Inventário dos Recursos do Ambiente Familiar (RAF), o Critério de Classificação Econômica Brasil (CCEB), o desempenho escolar e dados sociodemográficos dos participantes – idade, sexo, ano escolar em curso e escolaridade parental.

A distribuição da amostra do presente estudo pode ser considerada equilibrada em relação às variáveis sexo, ano escolar, idade e desempenho escolar. É importante ressaltar que a maioria dos participantes deste estudo era pertencente à classe social A e declarou que o/a chefe da família possuía Ensino Superior completo como nível de instrução – cenário que não retrata o panorama nacional, no entanto, corrobora dados referentes às classes sociais de maior incidência nesta pesquisa^(9)^.

O histórico acadêmico dos pais, incluindo reprovações e anos de estudo, é um fator que exerce influência sobre o desempenho acadêmico dos filhos^(9,13-15)^. A maior escolaridade parental aumenta as chances de melhor acesso a estratos sociais mais elevados, o que influencia na disponibilidade de recursos e, consequentemente, no desempenho escolar do adolescente, na frequência e no tipo de atividade de lazer, além de interferir na prática ou não de outras atividades extraescolares^(9)^. Este processo produz um ciclo em que os adolescentes cujas famílias têm melhor condição socioeconômica e maior escolaridade possuem mais recursos e pais com melhores condições de participar da vida escolar de seus filhos, ao passo que a renda das famílias em situação de vulnerabilidade social são direcionadas para o sustento, com pouco orçamento disponível para o lazer^(9)^.

Foi verificado que apenas o eixo presença de jornais e revistas não foi contemplado, portanto é possível questionar primeiramente o acesso aos recursos ambientais das classes sociais A e B. A literatura indica que a distribuição de recursos é afetada pela condição socioeconômica, como demonstrado no acesso a passeios, a brinquedos e a livros – situações associadas a maior conforto, este sinalizado pela presença de comodidades no lar^(16)^. O tópico presença de jornais e revistas do RAF não é mais compatível com o modo de consumo atual de informações, portanto a pequena expressão de pais que admitiram possuir jornais e revistas fisicamente disponíveis em domicílio realça a migração para o consumo digital de reportagens e de notícias ocorrida desde a elaboração do instrumento. De acordo com dados do Instituto Verificador de Comunicação (ICV)^(17)^, a circulação de jornais e revistas em formato digital tem aumentado, ao passo que o consumo impresso sofre quedas.

Diante do fato de que a maioria da amostra deste estudo obteve desempenho escolar classificado como excelente ou bom, embora a amostragem tenha sido aleatorizada, é possível inferir que este resultado deve-se: ao fato de que os pais que aceitaram participar da pesquisa foram os mais interessados e que melhor acompanham seus filhos; ou ao perfil da escola e ao seu método de acompanhamento; ou ao melhor acesso a recursos das classes sociais A e B, o que poderia influenciar positivamente o desempenho escolar e impactado nos resultados. Foi observado que pessoas em vulnerabilidade social possuem menor disponibilidade de recursos e que o contexto familiar pode causar desajustes e interferir negativamente no desempenho escolar^(1)^.

As associações encontradas entre a participação dos pais em experiências estimuladoras, a idade e o ano escolar; as *práticas parentais que promovem a ligação família-escola*, a idade e o ano escolar; o RAF total, a idade e o ano escolar evidenciam mudanças na interação dos adolescentes com os pais e com as tarefas no decorrer da trajetória escolar. As correlações positivas de magnitude fraca observadas entre os passeios realizados nos últimos doze meses, a idade e o ano escolar corroboram estudo prévio, onde a relação entre a família, a escola e os recursos oferecidos pelos pais, bem como a forma de utilizá-los, muda com a idade^(18)^.

A realização de atividades fora da escola apresentou correlação positiva de magnitude fraca com outros tópicos do RAF, evidenciando a importância de dois elementos: a presença dos pais e a disponibilidade de recursos. Esta relação também ficou evidente na interação entre as atividades regulares programadas e a existência de livros com os pais e os passeios realizados nos últimos doze meses. Tais resultados podem ser indicativos de que os passeios representam uma parcela das atividades realizadas em família.

O tópico passeios do último ano também apresentou uma correlação positiva de magnitude fraca com as atividades regulares programadas e com a disponibilidade de livros, revistas e jornais em casa. A existência de uma correlação positiva de magnitude moderada entre os brinquedos e as atividades com os pais, e a correlação positiva de magnitude fraca entre atividades com os pais e atividades regulares programadas, brinquedos e livros pode ser atribuída não somente à oferta dos objetos, mas também à interação dos pais com os filhos^(9,19)^. Pesquisa indica que ter livros, brinquedos pedagógicos disponíveis e contar com o acompanhamento parental nos afazeres escolares foram fatores relacionados a um melhor desempenho escolar^(9)^.

A importância da interação com os pais, da disponibilidade de brinquedos e livros, do bom aproveitamento do tempo livre, do acesso a atividades programadas de aprendizagem, além de materiais que estimulem o raciocínio, torna-se evidente ao analisar a influência destes aspectos nas pontuações brutas dos demais domínios. Alunos com desempenho escolar satisfatório têm mais momentos de interação com seus pais, possuem livros em casa, realizam atividades de lazer e mantêm uma rotina fixa estabelecida^(19)^.

É fundamental salientar que o estudo explicitou a correlação positiva de magnitude fraca entre a disponibilidade de livros e o desempenho escolar médio dos participantes. Tais dados reforçam a ideia de que o maior volume de recursos do ambiente familiar – com destaque para a existência de livros ao alcance dos adolescentes – tem influência positiva no desempenho escolar^(9,19)^.

A análise entre o desempenho escolar, a pontuação total obtida no Inventário dos Recursos do Ambiente Familiar (RAF total) e seus três domínios (processos proximais, ligação Família-Escola e práticas parentais que sinalizam algum grau de estabilidade) revelou correlações importantes. Foi encontrada correlação positiva de magnitude forte entre o RAF total e os domínios: *recursos que promovem processos proximais* e *práticas parentais que promovem a ligação família-escola*. Tais resultados evidenciam a importância que a presença dos pais e a participação destes traz para a vida escolar de seus filhos. O domínio das *atividades previsíveis que sinalizam algum grau de estabilidade na vida familiar* também revelou uma correlação positiva com o RAF total, porém de magnitude moderada.

Houve, ainda, correlação positiva fraca entre o domínio *práticas parentais que promovem a ligação família-escola* com os domínios *recursos que promovem processos proximais* e *atividades previsíveis que sinalizam algum grau de estabilidade na vida familiar*. É importante ressaltar que uma relação saudável entre a família e a escola é significativa e benéfica para o processo educativo^(9)^, posto que esta relação pode influenciar os *recursos que promovem processos proximais* ^(20,21)^.

A correlação positiva forte observada entre o RAF total, os *recursos que promovem processos proximais* e a *práticas parentais que promovem a ligação família-escola*; e a correlação positiva moderada entre as *atividades previsíveis que sinalizam algum grau de estabilidade na vida familiar*, o RAF total e o desempenho escolar; reforçam que a rotina e a presença de atividades com os pais, bem como a presença de livros, são elementos que refletem no desempenho escolar do estudante^(16)^. Outro elemento que parece influenciar as notas obtidas foi a presença de livros, que mostrou uma correlação positiva de magnitude fraca com o desempenho acadêmico. Existem evidências de que as práticas parentais possam ter influência sobre o desempenho em leitura de palavras não frequentes, e que os diálogos entre pais e filhos viabilizam a ampliação dos conhecimentos^(22)^.

Os achados da investigação relatada neste texto apontam que a classe social, a escolaridade parental e os recursos presentes no ambiente familiar – principalmente a disponibilidade de livros – são fatores que influenciam no desempenho escolar. Além disso, é possível inferir que a participação dos pais em experiências estimuladoras, no estabelecimento de rotinas e a manutenção de uma boa relação entre a família e a escola também são essenciais para um bom resultado acadêmico.

É importante ressaltar que os resultados deste estudo devem ser analisados com cautela, pois remetem apenas à população de referência, ou seja, adolescentes do Ensino Fundamental II em um contexto específico; portanto, não são passíveis de generalização. Outra reflexão que deve ser feita é a de que não existe uma relação direta entre o mau desempenho e a falta de recursos, uma vez que as notas obtidas pelos alunos podem ser influenciadas por outras variáveis, como a forma de avaliação da instituição e a abordagem de ensino adotada. Sendo assim, é necessário que haja outros estudos que investiguem a relação dos recursos do ambiente familiar com o desempenho escolar em cenários diversos para esclarecer melhor como essas associações e relações ocorrem.

Esta pesquisa apontou avanços no que diz respeito às relações entre os recursos do ambiente familiar, os fatores sociodemográficos, a escolaridade parental e o desempenho em um contexto diferente do habitualmente encontrado na literatura. Também apontou uma correlação dos passeios realizados no último ano com as atividades compartilhadas com os pais e com os brinquedos.

## CONCLUSÃO

A análise dos dados mostrou que existe uma associação entre a idade e o ano escolar com a *práticas parentais que promovem a ligação família-escola* e com os *recursos que promovem processos proximais* e foram analisadas as correlações entre os recursos do ambiente familiar, os dados sociodemográficos e o desempenho escolar dos participantes. A pesquisa revelou a importância da relação positiva entre a família e a escola, da realização de passeios com os pais, do acesso a brinquedos e da presença dos livros em casa para o desempenho escolar dos adolescentes.
